# Comprehensive Two-Dimensional Gas Chromatography as a Bioanalytical Platform for Drug Discovery and Analysis

**DOI:** 10.3390/pharmaceutics15041121

**Published:** 2023-03-31

**Authors:** Atiqah Zaid, Norfarizah Hanim Hassan, Philip J. Marriott, Yong Foo Wong

**Affiliations:** 1 Centre for Research on Multidimensional Separation Science, School of Chemical Sciences, Universiti Sains Malaysia, Penang 11800, Malaysia; 2 Australian Centre for Research on Separation Science, School of Chemistry, Monash University, Wellington Road, Clayton, Melbourne, VIC 3800, Australia

**Keywords:** GC×GC, metabolomics, drug discovery and development, mass spectrometry, biomarkers, tuberculosis, cancer, COVID-19, psychiatric disorder

## Abstract

Over the last decades, comprehensive two-dimensional gas chromatography (GC×GC) has emerged as a significant separation tool for high-resolution analysis of disease-associated metabolites and pharmaceutically relevant molecules. This review highlights recent advances of GC×GC with different detection modalities for drug discovery and analysis, which ideally improve the screening and identification of disease biomarkers, as well as monitoring of therapeutic responses to treatment in complex biological matrixes. Selected recent GC×GC applications that focus on such biomarkers and metabolite profiling of the effects of drug administration are covered. In particular, the technical overview of recent GC×GC implementation with hyphenation to the key mass spectrometry (MS) technologies that provide the benefit of enhanced separation dimension analysis with MS domain differentiation is discussed. We conclude by highlighting the challenges in GC×GC for drug discovery and development with perspectives on future trends.

## 1. Introduction

Drug discovery and development have become challenging over the years due to the continuous demand for appropriate strategies aimed at effectively treating diseases or clinical conditions [1,2,3,4]. Despite the critical need for new and improved drugs, the current process is arduous and expensive, not to mention the high risk of failure. It has been globally accepted that bioanalysis, conceptually termed as the quantitative measurement of biomarkers, chemical entities, biologics, and/or their metabolites in biological matrixes, plays a pivotal role in understanding the pharmacological and toxicological properties across all phases in the drug development pipeline (i.e., initial research, drug discovery, preclinical development, nonclinical investigation, clinical trial, and post-approval studies) [5,6]. Indeed, numerous bioanalytical strategies (e.g., sample preparation, method development, and validation) are constantly progressing toward achieving high-throughput analyses and rigorous characterization, particularly when dealing with complex biological specimens (e.g., blood, feces, hair, plasma, saliva, serum, tissues, and urine) intended for diagnosis, prognosis, and treatment of diseases or clinical conditions [7,8,9,10,11,12,13].

The contribution of ‘-omics’ technologies is massive in bioanalytical research in which genomics, transcriptomics, proteomics, and metabolomics have long been recognized as cost- and time-efficient approaches in implementing the search for critical biomarkers [14,15]. Among these, metabolomics-based strategies, which involves high-throughput identification and quantitative analysis of small molecules (<1500 Da) in the metabolome [16], are gaining wider attention over the last decade owing to the ability to facilitate a better understanding of complex phenotypes in diverse biological systems. While gene and protein expressions predict cell functioning, metabolites are much closer to phenotypes and hence possess the ability to closely reflect the actual cellular activities or environments [17,18]. Metabolomics allows deeper insights into the alteration of crucial metabolites by internal and/or external stimuli (e.g., drug administration, environment, and disease progression) in contrast to the other ‘-omics’. To date, over 200,000 metabolites have been tabulated in the Human Metabolome Database (HMDB) v.5 [19], highlighting the notable complexity of metabolic profiles in diverse biological systems. The presence of drugs (and their metabolites) at low concentration levels as well as the inherent complexity of biological samples further complicate the analysis [20,21]. Nevertheless, technological advances in various chromatographic techniques (e.g., liquid chromatography (LC), supercritical fluid chromatography (SFC), and gas chromatography (GC)), along with improved detection capabilities, have rendered the discovery and analysis of pharmaceutical compounds possible, at trace levels that were unattainable several years ago [22,23,24].

GC is well established as an effective tool of choice for the separation of low molecular weight (semi)volatile chemical entities, where the column stationary phase and dimensions serve as the main aspects governing chromatographic efficiency and resolution [25,26]. Since the invention of the capillary column by Golay, numerous developments (e.g., column type/phase and coating techniques) have taken place over the years owing to the breakthrough in the separation power and inertness (as compared to packed column), which subsequently led to countless applications of GC in many fields such as petrochemicals [27,28,29], foods [30,31,32,33,34], plants [35,36,37,38], pharmaceuticals [39,40,41,42,43,44] and others [45,46,47,48]. Despite the versatility and high separation power of the technique, classical one-dimensional (1D) GC poses the limitation of not sufficiently disentangling critically co-eluted components due to its limited peak capacity and overwhelming number of distinct components with high chemo-diversity in real-life biological samples. While the use of deconvolution procedures in GC coupled with mass spectrometry (MS) may be useful for the identification of overlapping components, the outcome is very much subject to the uniqueness of the components’ individual spectra [49]. Consequently, the demand for greater peak capacity to resolve these intricate mixtures of molecules gave impetus to further developments. The advent of multidimensional GC presents an opportunity to address the issues of sample dimensionality by expanding the separation space, along with increasing resolving power. This review will briefly provide an overview of the multidimensional GC (MDGC) techniques with a particular focus on the current advances in state-of-the-art comprehensive two-dimensional GC (GC×GC). Recent applications of GC×GC in bioanalytical studies covering disease-related biomarkers and host response to drug treatment published within the period of 2013−2022 will be reviewed. Moreover, challenges and future perspectives of GC×GC in pharmacometabolomics will be discussed. 

## 2. Multidimensional Gas Chromatography

MDGC is a high-performance separation technique that ideally employs the combination of two (or more) ‘orthogonal’ columns—implying they should have distinctly different selectivity toward analytes—which are connected in a sequential fashion via interfacing devices [50,51]. MDGC can be implemented using two main experimental arrangements, namely (i) the classical heart-cutting (H/C) MDGC; and (ii) GC×GC. The first application of H/C MDGC was demonstrated by Simmons and Synder in 1958, where selected portion(s) of eluents (or heart-cut(s)) were transferred using a switching valve from the first-dimension (^1^D) column into second-dimension (^2^D) column of disparate selectivity for additional separation [52]. In 1968, Deans then pioneered the microfluidic flow-switching mechanism that we know today as the Deans’ switch, which has brought a lasting and substantial impact on the practice of MDGC [53]. It was commemorated in a paper by Sharif et al. after a half-century of progress, innovations, and applications [54]. Several other innovations have also been developed (e.g., cryogenic trapping and double ovens) to further enhance the quality of separation in MDGC [55,56]. As discussed by Giddings [57], the concept of multidimensionality involves the requirement of two conditions: (i) the analytes present in a sample should be subjected to two or more independent separation mechanisms (i.e., columns’ selectivity); and (ii) the resolution of analytes achieved in the ^1^D separation is preserved until the whole separation process is complete. Although the separation space is now expanded, H/C MDGC, as described here, cannot be regarded as a truly ‘comprehensive’ separation system due to the incapability of providing maximum resolution to the entire sample components. In cases where many peaks of interest demand critical separation across the whole sample, multiple heart-cut samplings are required to acquire a full-spectrum analysis of a sample, which may cause a dramatic shift/increase in the total analysis time [58]. Notwithstanding its limited application, H/C MDGC is still considered a prime choice for target analysis. Further details on H/C MDGC have been discussed previously in several reviews [49,59,60,61,62,63] and will not be reiterated here. This brief summary of H/C MDGC serves only as a prelude to the current knowledge of GC×GC.

## 3. Comprehensive Two-Dimensional Gas Chromatography

In retrospect, GC technology has undergone one of the most outstanding breakthroughs, with the introduction of GC×GC using resistively-heated modulation by Liu and Phillips in 1991, which fully demonstrated the concept of comprehensiveness in multidimensional separation [64]. Since then, the research interest in GC×GC bloomed with many fundamental studies and extensive reviews reported on its early developments and various applications [61,62,63,65,66,67,68,69,70,71,72]. Today, it has been widely recognized that GC×GC significantly outperforms 1D GC on peak capacity, providing peak capacities of >20,000 [73], which can essentially translate to superior separation performance for the analysis of complex chemical entities.

### 3.1. Technical Implementations

From a technical perspective, GC×GC can be considered an extension of MDGC approaches, where multiple heart cuts are continuously sampled from a conventional ^1^D column at a defined modulation period (*P*_M_), into a short ^2^D column for additional separation, throughout the entire analysis. An interfacing device, commonly known as a modulator, is specially configured to periodically sample, re-focus and re-inject contiguous ‘slices’ of ^1^D fractions rapidly into the ^2^D column. Ideally, the separation in ^2^D should be completed before the injection of the next ^1^D fractions, thus avoiding the recombination of ^1^D component separation in ^2^D. The present-day modulation systems can be broadly categorized into two types, namely thermal and valve-based (including flow). A thermal modulator employs temperature control, either using a refrigerant unit or a cryogen to trap analytes and introduce them into the ^2^D via rapid heating. A valve-based modulator utilizes gas flow for the control and isolation of portions of the ^1^D eluate before redirecting them for ^2^D separation, in which case a longer ^2^D is used [74]. Among these, thermal modulation systems appear to be more prevalent due to a greater degree of sensitivity enhancement and better modulation performance [74]. A major disadvantage of thermal modulation is the high consumption of cryogen (e.g., liquid nitrogen and liquid carbon dioxide) to trap and focus volatile components, as well as the bulkiness of the instrumentation [75]. Recent innovations in GC×GC modulator technology are moving toward simpler and more economical devices. Currently, a few notable studies have demonstrated the practicability of cryogen-free thermal modulators that provides relatively good portability and low operational cost compared to cryogenic modulators [76,77,78,79,80,81,82,83,84]. For instance, the solid-state modulator (SSM) employs mica-thermic heating and thermoelectric cooling (also known as Peltier cooling) on a mechanically moving modulation column that continuously oscillates back and forth between the hot and cold zones, achieving modulation without the need for cryogens [77]. Duty cycle, injection pulse width, *P*_M,_ and resulting peak capacity in ^2^D separation are among the important parameters in gauging the performance of modulation [74]. The development, advantages, and shortcomings of various modulation systems have been comprehensively reviewed and can be found elsewhere [74,85,86,87]. 

In order to ensure reliable reconstruction of the ^1^D separation, analysts need to define a sufficient number of modulations across the ^1^D peak, which can be defined using the term modulation ratio (*M*_R_; peak ^1^*w*_b_ divided by *P*_M_) [88]. Khummueng et al. imply that an *M*_R_ of 3.0 is sufficient for quantitative measurement, while an *M*_R_ ~1.5 is sufficient for qualitative analysis [88]. For practitioners, the *P*_M_ settings need to be properly selected to avoid the phenomenon of over- or under-sampling that would potentially compromise the integrity of the 2D separation. A relatively short *P*_M_ (i.e., large *M*_R_ values) might reduce the solute’s detectability and increase the tendency of wraparound, while a longer *P*_M_ generates fewer modulation events that degrade the ^1^D separation in addition to potentially overloading the ^2^D column [89]. A typical GC×GC arrangement requires two columns to be interfaced through the modulator, with the outlet of the ^2^D column connected to a detection system. In this instance, analysts should carefully decide the stationary-phase combinations that act as the basis to enhance chemical selectivity. It is worth noting that the stationary-phase chemistry governs the strength and types of mass transfer interactions (e.g., dispersive interactions, dipole-dipole, dipole-induced dipole interaction, etc.) with the solutes, and the Abraham solvation parameter model has been extensively used to characterize such interactions [90,91,92]. Ionic liquid (IL) phases represent new classes of novel GC stationary phases that offer additional selectivity for the separation of polar and nonpolar molecules [93], which currently remain underexplored for GC×GC-based metabolomic studies. The high polarities of IL phases are expected to benefit the analysis of a wide range of polar metabolites and will be useful in adjusting the phase selectivity differences for ^1^D and ^2^D. For practitioners, the degree of orthogonality can be used as a guideline for the selection of column sets to maximize the utilization of the 2D separation space, which ideally translates to better-resolving power. A few metrics have been reported as potential measures of orthogonality of GC×GC separations [94,95,96,97,98,99]. Apart from deducing the appropriate stationary-phase chemistries for ^1^D and ^2^D, the elution of all pulsed peaks in ^2^D should be completed within the selected *P*_M_ to avoid wraparound, and such requirements necessitate the use of fast-elution short narrow-bore columns as the ^2^D when cryogenic modulators are used. Several comprehensive reviews have summarized the GC×GC column selection and optimization strategies and will not be reiterated here [50,100,101,102]. 

The very narrow chromatographic bandwidths generated by ^2^D apply constraints upon the types of detectors, necessitating fast data acquisition speed. For modulated peaks with ^2^*w*_b_ of 100 ms, a detector sampling rate of ≥100 Hz is required for the reliable construction of a Gaussian peak, which is best afforded by the universal flame ionization detector (FID). Kueh et al. demonstrated the first application of GC×GC–FID for the screening of illicit drugs and their metabolites in biological fluids [103]. While providing a sufficiently fast acquisition rate (up to 500 Hz), a FID is unable to provide descriptive information, which reduces its applicability for bioanalysis, especially for untargeted metabolic profiling of biological samples. The coupling of GC×GC to MS remains the preferred option for bioanalysts due to its informing power, facilitating molecular structure identification and confirmation. The use of GC×GC with quadrupole MS (QMS) with a reduced mass scanning range designed to maximize scan frequency (42–235 Da; approximately 20 Hz) for the profiling of underivatized drugs was first demonstrated by Song et al. [104]; the relatively low sampling rate of QMS in full scan mode renders it less ideal for untargeted screening purposes. Recently, a few studies have demonstrated the applicability of GC×GC with triple quadrupole MS (MS/MS) for targeted analysis, illustrating a notable improvement in detection sensitivity and selectivity [105,106,107]. However, GC×GC–MS/MS operated in MRM modes have not yet been evaluated for quantitative bioanalytical studies. On the other hand, time-of-flight MS (TOFMS), capable of attaining high acquisition speeds (up to 200 Hz for high-resolution TOFMS and 500 Hz for low-resolution TOFMS) without spectrum distortion, has been widely used as the MS of choice for GC×GC analysis [108,109]. The hyphenation of GC×GC to various MS for a wide range of metabolomics studies has been recently reviewed [67,110,111]. Numerous studies have succinctly demonstrated the applicability of GC–high-resolution mass spectrometry (GC–HRMS) for the screening of drugs and their metabolites in biological matrixes [112,113,114,115,116,117]; however, the use of GC×GC–HRMS for bioanalytical studies remained underexplored. HRMS such as accurate mass TOFMS (accTOFMS), Fourier Transform Ion Cyclotron Resonance MS (FTICRMS), and Orbitrap technologies are capable of providing high accuracy *m/z* measurements (typically within 0.001 amu) with mass accuracy of less than 5 ppm [118,119]. This feature can greatly advantage elemental formula prediction for unknowns and improve the accuracy of library matching for metabolite annotation and identification. However, the low data acquisition rate of Orbitrap and FTICRMS limits their applicability due to the associated difficulties in collecting sufficient data points for an accurate reconstruction of narrow modulated peaks. With continual improvement in data acquisition speed, the hyphenation of GC×GC to HRMS is expected to flourish as a “super-resolution” analytical tool that can generate a wealth of informative data in a single analysis for complex biological samples. It is anticipated that the scope of GC×GC–HRMS applications will soon be expanded to drug metabolism research, harnessing its unrivaled chromatographic separation and MS-informing power.

### 3.2. Data Acquisition and Analysis

**Figure 1:**
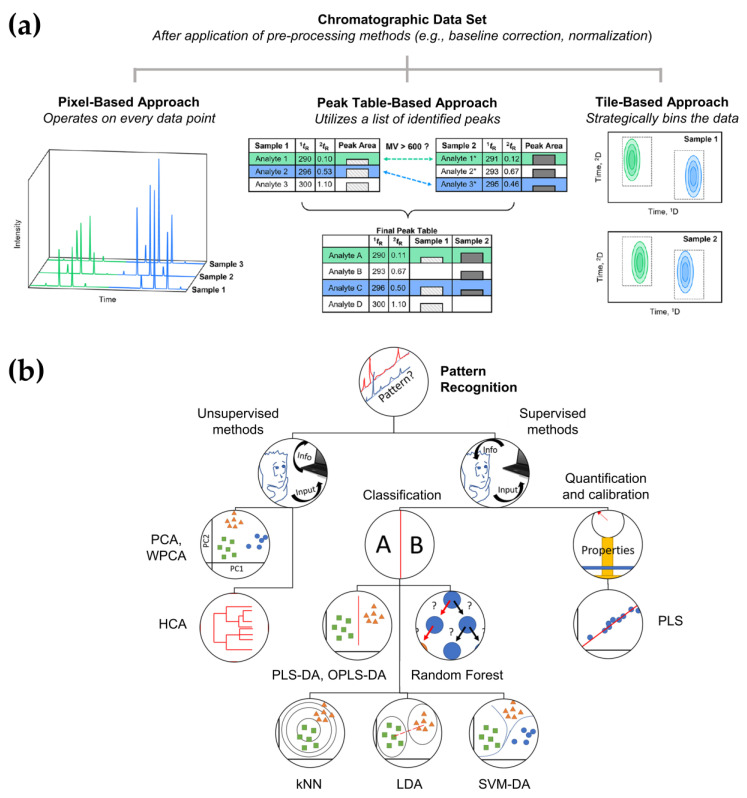
Application of several GC×GC data pre(processing) methods: (**a**) retention time alignment approaches, adapted with permission from Ref. [123]. Copyright © 2023 by the authors; American Chemical Society, Washington, DC, United States; and (**b**) chemometrics techniques, adapted with permission from Ref. [131]. Copyright © 2023 by the authors; Elsevier B.V., Amsterdam, The Netherlands.

Continuing advancement in MS detection methods has enabled the handling of high detector acquisition frequencies (50 to 200 Hz) to accommodate the narrow peaks generated by ^2^D in GC×GC [120]. Such a high sampling rate results in the generation of high-dimensional data sets and data files up to several hundred gigabytes for HRTOFMS, complicating manual data manipulation. Clearly, there remains a need for robust and reproducible data analysis strategies, possibly with a reduced intervention of the analyst, to take advantage of the massive data. Chemometrics has therefore been applied to the interpretation and optimization of analytical methods by applying mathematical and computational methods to extract relevant information from chemical or process data [121]. Since the compounds of interest are known a priori in targeted analysis, deconvolution or decomposition approaches would suffice to mathematically separate, identify, and quantify overlapping analytes [122,123]. In cases where the coelution of target analytes is critical, more sophisticated data handling may be desirable. Untargeted analysis, on the other hand, contains unknown analyte composition or compounds of interest, and thus the analysis is much more complex. While any classical GC software can be used for data acquisition, specific software (commercial or open-source) such as ChromaTOF, Canvas-2DGC, OpenChrom, and Guineu is required for the reconstruction of signal and generation of two-dimensional plots or data [120]. These signals are then pre-processed to correct various artifacts (e.g., missing values (or data), noise, baseline shifts, multiplicative effects, and peak shifts) and assist in the accurate prediction of chemically-relevant features [124]. It should be noted that this step carries the risk of eliminating only certain types of artifacts as well as potentially useful information. Defined as the lack of one or more entries in the matrix containing the experimental data, missing values are often mishandled in data preprocessing and may give rise to severely biased results within pharmaceutical research as well as diminished statistical power [125,126,127]. Davis et al. have recently reviewed several imputation strategies to address various types of missing values in GC×GC data [127]. The incorporation of artificial intelligence (AI) in analytical chemistry has allowed ground-breaking advances, especially in the pharmaceutical industry, where it is anticipated to assist in producing quicker, cheaper, and more effective drug discovery and development technologies [128,129]. Automated data processing approaches, including machine learning, feature selection, discriminant analysis, and regression analysis, have been continually evolving to address problematic variations across multiple GC×GC chromatograms. More recently, the integrated implementation of data alignment approaches (pixel-based, peak table-based, and tile-based) with chemometrics in software packages have been described in detail, enabling simpler extraction of information with minimal user impact (Figure 1) [123,130]. Given that data analysis typically entails a number of integrated approaches, there is not yet any evaluation tool or data processing workflow available that can be deemed as the most ideal for gleaning the required information. Further details and drawbacks on data acquisition, pre-processing, and analysis of GC×GC have been comprehensively reviewed elsewhere [120,123,130,131,132,133].

## 4. Applications of GC×GC in Drug Discovery and Analysis

**Figure 2:**
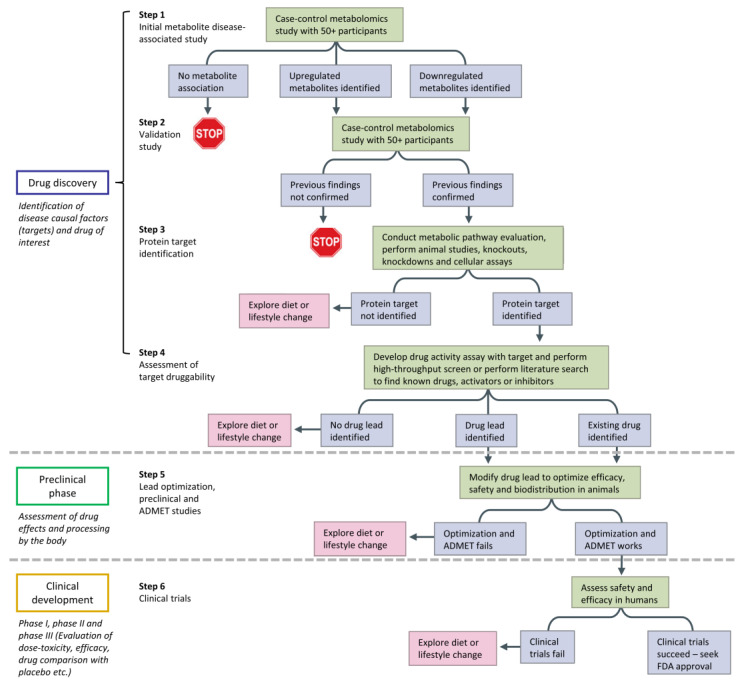
A decision tree for metabolite-based drug discovery and development. ADMET, absorption, distribution, metabolism, excretion, and toxicity; FDA, US Food and Drug Administration, adapted with permission from Ref. [136]. Copyright © 2023 by the authors; Nature Publishing Group, London, England.

The discovery and development of new drugs and treatment strategies generally commence with the establishment of targets and/or the understanding of the underlying disease mechanisms responsible for the etiology and pathological progression in biological systems [4,134]. The whole process, starting from early academic exploration to regulatory approval and use, is lengthy and expensive as it takes around 10–15 years or longer, with an average cost of over $1–2 billion [4,135]. Despite the emergence of many advanced technologies by which clinical drug discovery and development pipelines find their way into many scientific disciplines (e.g., artificial intelligence, biomedical, pharmaceutical, etc.), its high attrition rate during the process remains a key issue. Metabolomics is now leading to a paradigm shift in the discovery, development, delivery, and dosage of drugs, with the advantage of being a cheap, rapid, and sensitive option [136,137]. Figure 2 illustrates how metabolomics-based approaches may be performed in the discovery and development of new therapeutics. By gaining new metabolic insights into pathogenesis, especially using GC×GC, drug targets, and leads could then be identified and assessed to shed further light on individualized therapeutic intervention. Although its application is quite recent in this field, GC×GC has demonstrated significant potential in improving the quality of drug research and development. Selected clinical and pharmacological studies involving disease-related biomarkers and host response to drug treatment in the last 10 years will be discussed in this section, with additional details summarized in Table 1.

### 4.1. Tuberculosis

Tuberculosis (Tb) is one of the leading causes of death due to the infection of a bacterial pathogen, *Mycobacterium tuberculosis,* with common symptoms such as bad cough with mucus, pleurisy, hemoptysis, dyspnea, wheezing, weakness/fatigue, weight loss, loss of appetite, chills/fever, and night sweats [156]. According to World Health Organization (WHO), approximately 10.6 million people suffered from Tb, with 1.6 million deaths reported in 2021 [157]. The first-line Tb therapy under the Directly Observed Treatment Short-Course (DOTS) as a Tb control strategy, recommended by WHO, includes a six-month regimen with isoniazid, rifampicin, pyrazinamide, and ethambutol. Previous efforts have been made by researchers, particularly Loots’ group, in which the bacterial strain [138,142,143,144], as well as urine samples of Tb patients [139,140,141], were subjected to GC×GC to further understand the disease mechanism and elucidate the roots of Tb treatment failure.

Poor adherence of Tb patients to the prescribed drug treatment causes an alarming prevalence of multidrug resistance, where such resistance arises from extended treatment duration as well as the generation of adverse side effects [143]. Multidrug-resistant Tb is declared a health security issue as only one in three drug-resistant Tb patients accessed treatment in 2020 [157]. The earliest work of GC×GC on Tb was reported by Loots in 2014, where the metabolomic profiling of two different isoniazid-resistant cultured strains of *M. tuberculosis* (H15 and H71) and wild-type TB72 parent (control) strain was performed using GC×GC–TOFMS [138]. Monoresistance to isoniazid was stated as the most common form of drug resistance in Tb patients. The use of principal component analysis (PCA) led to the identification of 23 biomarkers capable of discriminating between the three strains. The majority of the compounds (alkanes, alcohols, fatty acids, and compounds related to a direct adaption to oxidative stress) were observed to be at the comparatively elevated level in both isoniazid-resistant strains as opposed to the wild-type parent strain. As a result, the current study was able to provide a more holistic elucidation of the compensatory mechanisms linked to *katG* mutation and the resulting isoniazid resistance in *M. tuberculosis* through metabolic pathways and metabolites involved, complementing the mechanisms proposed by various genomic and proteomic studies, which were not completely understood. This study pioneered research on the exploration of the metabolomics of drug-resistant strains that led to Tb treatment failure. Subsequently, Luier and Loots applied GC×GC–TOFMS to evaluate the metabolomic profiles of urine samples from Tb-positive and -negative groups [139]. Out of 507 compounds detected, 12 urinary Tb metabolite markers were discovered to distinguish these two subjects. The presence of unique biomarkers was induced by the adaptation of the host metabolome and/or host-pathogen interactions. Such alteration led to abnormalities in the host’s metabolism of fatty acids and amino acids (i.e., tryptophan, phenylalanine, and tyrosine), resulting in a metabolite profile similar to that of phenylketonuria patients. This study sheds light on the mechanism of the abnormal symptoms related to Tb and offers potential avenues for the development of more effective treatment methods.

The capacity of metabolomics via utilization of GC×GC–TOFMS to predict prognosis from monitoring Tb progression in the early phase of the DOTS treatment regimen was also demonstrated by Loots’s group [140,141]. In 2017, the differentiation of urine metabolites was observed in successful (*n* = 26) and unsuccessful (*n* = 15) treatment groups from PCA scores plots, using urine samples collected at the time of diagnosis and after treatment at week 26 [140]. Following the aim of the study to identify biomarkers for the early prediction of treatment response, only the former PCA result was further evaluated, which resulted in 50 characteristic metabolite markers that best discriminate between both treatment outcome groups. The Tb-treatment-failure group was characterized by the upregulation of metabolites associated with an imbalance in the gut microbiome. This study also corroborates the previous finding [139] as the altered amino acid metabolism was observed and confirms its associations to the increased interferon-gamma due to the host’s immune response to *M. tuberculosis* and a compromised insulin secretion mechanism. In the same year, the group performed a statistical analysis approach based on logistic regression in which 18 univariate urine metabolite markers (absolute fold change > |2|; Mann–Whitney *p*-value ≤ 0.05; and an effect size > 0.3) of Tb patients were considered as possible candidates to predict the treatment outcome at the time of diagnosis, prior to the first-line Tb treatment [141]. Based on the forward logistic regression model, two metabolites (3,5-dihydroxybenzoic acid and 3-(4-hydroxy-3-methoxyphenyl)propionic acid) were identified as the markers of treatment failure with acceptable fit by a nonsignificant Hosmer–Lemeshow statistic (*p* = 0.444). From the mechanistic biological perspective, both metabolite markers are associated with a microbial-derived microbial imbalance, causing an alteration of the microbiome in Tb patients, which then leads to the reduced efficacy of the anti-Tb drugs. However, a larger sample cohort is required to further validate and develop the predictive treatment model for Tb, as the possible mechanism contributing to the treatment failure remains unknown. These studies [140,141] highlight the significant advantage of the application of GC×GC in metabolomics, the function of which is to provide molecular insights into early diagnosis, new drug development clinical trials, and individualized patient treatment.

The high prevalence of multidrug resistance in Tb patients emphasizes the need for the research and development of new anti-Tb drug lines and an alternative treatment strategy. Nonetheless, approval of a new drug will have to undergo a lengthy drug trial phase; thus, repurposing an existing approved drug will be feasible. Following the previous findings, Loots’ group has extended their research by examining the pharmacometabolomics of selected drugs for the treatment of Tb. Colistin sulfate, a polymyxin antibiotic, was reported to display significant antimicrobial activities against several mycobacteria (*M. avium, M. aurum, M. xenopi,* and *M. smegmatis*), suggesting similar activities against *M. tuberculosis*. Thus, the antimicrobial mechanisms of colistin sulfate on *M. tuberculosis* were assessed using GC×GC–TOFMS to identify the metabolite markers of *M. tuberculosis* upon treatment with colistin sulfate [142]. The multi-statistical approach revealed 21 key markers that effectively distinguished between individually cultured *M. tuberculosis* in the presence and absence of colistin sulfate, of which 15 markers account for the elevated fatty acid biosynthesis and cell wall synthesis. These metabolite markers indicated the upregulation of fatty acid synthesis pathways for cell wall repairing, thus verifying the antimicrobial mechanism of colistin sulfate inducing structural disruption of *M. tuberculosis* cell wall.

Recently, ciprofloxacin, a fluoroquinolone antibiotic originally used to treat urinary tract infections, was considered a candidate for anti-Tb therapy. Although ciprofloxacin is less potent compared to other fluoroquinolones (e.g., moxifloxacin and levofloxacin), the antibiotic demonstrated the highest drug clearance rate and minimal adverse drug reactions (≤5%) [158,159]. Thus, the metabolite profile of ciprofloxacin-treated *M. tuberculosis* and the controls were analyzed with GC×GC–TOFMS to further elucidate the mechanism of action against *M. tuberculosis* [143]. A total of 26 key markers were identified to best describe the variation between ciprofloxacin-treated and control subjects, with 61.5% (16 out of 26 markers) elevated, which are predominantly fatty acids. Ciprofloxacin treatment on *M. tuberculosis* observed prominent alterations in several pathways associated with the cell wall and DNA repair mechanisms. This study provides a better understanding of ciprofloxacin’s mode of action and demonstrates the *M. tuberculosis*-induced shift to the non-replicative phase, which is a key mechanism in determining its persistence and tolerance to various anti-Tb drugs. Within the same year, Knoll et al. applied a GC×GC–TOFMS approach to evaluate the metabolic alterations of *M. tuberculosis* treated with decoquinate derivative RMB041 in comparison to non-treated *M. tuberculosis* controls [144]. Decoquinate is an anticoccidial quinolone, generally used as a broad-spectrum antibiotic that has recently exhibited promising antimicrobial activity against *M. tuberculosis* (MIC90 = 1.61 µM), with low cytotoxicity and excellent pharmacokinetic characteristics [160]. Based on compliance with the following criteria: (PLS-DA variable importance in projection (VIP) value > 1; *p*-value < 0.05; effect size > 0.8), 36 metabolites were selected as markers discriminating between the decoquinate-treated *M. tuberculosis* and controls. Alterations in the metabolite profiles were observed concerning the drastic increase of fatty acids and glycerolipid precursors, amino acids, and urea cycle intermediates levels in decoquinate-treated *M. tuberculosis* culture. The authors suggested that decoquinate treatment led to the inhibition of protein synthesis and induced the state of dormancy in *M. tuberculosis,* whereby cell growth and division were ultimately inhibited. The implementation of a GC×GC metabolomic-based approach in these studies [142,143,144] facilitated the identification and elucidation of *M. tuberculosis* response mechanisms on the selected drug candidates, with the aim of repurposing existing approved drugs as potential anti-Tb therapy options.

### 4.2. Cancer

Dysfunctional metabolism is acknowledged as the hallmark of cancer, and multiple studies regarding the altered metabolism in cancer cells have been demonstrated [145]. In this respect, metabolomics plays a substantial role in the understanding of cancer metabolism and the identification of chemotherapeutic targets or lead. Recently, cancer stem cells have been considered a potential target for cancer treatment owing to their tumorigenicity and their critical roles in tumor metastasis, relapse, and chemo/radio-resistance [161]. Styczynski’s group studied the metabolite response of OVCAR-3 ovarian cancer cell lines (OCC) and isogenic ovarian cancer stem cell (OCSC) line toward therapeutic perturbation with docetaxel and three environmental perturbations (glucose deprived, hypoxia, and ischemia) using GC×GC–TOFMS [145]. Docetaxel is a potent chemotherapeutic agent in the taxane drug group that blocks tubulin depolymerization, leading to the inhibition of microtubule dynamics and cell cycle arrest [162]. Metabolite profiling of both cell lines putatively identified 44 metabolites from 177 analytes in OCC and 46 unique metabolites from 167 analytes in OCSC. Significant metabolic changes were observed due to docetaxel treatment in OCC, specifically in the amino acids and carbohydrates metabolic pathways, causing a drastic decline in the OCC growth rate within 48 h. In contrast, univariate analysis revealed no significant differences in the metabolites of the control and docetaxel-treated OCSCs. However, OCSC metabolism was slightly altered by docetaxel after 48 h, suggesting higher resistance of OCSC to docetaxel treatment compared to OCC. In the following year, GC×GC–TOFMS was utilized to profile the intracellular and extracellular metabolomes of both OVCAR-3 OCC and isogenic OCSC cell lines [146]. A total of 211 intracellular and 203 extracellular reproducibly measurable analytes were detected; of them, 40 and 46 unique potential biomarkers were identified in the intracellular and extracellular metabolites, respectively. Unsupervised dimensional reduction using PCA for the identified metabolites demonstrated a complete separation for the intracellular metabolites, revealing a distinct pattern of both OCC and OCSC. Amino acids and carbohydrates were higher in the OCC than in OCSC, while the presence of aliphatic compounds was observed to be lower in OCC. The notable differences in the metabolomic profile of these two cell lines were associated with several metabolic pathways, particularly arginine and proline metabolic pathways. These metabolic changes may contribute to the functional differences between OCSC and their more differentiated cells that represent the majority of bulk tumor tissue. Nevertheless, the information on the inherent differences in the metabolomics and drug response of cancer cells and cancer stem cells obtained from these studies could potentially be useful in the development of targeted treatments for cancer stem cells or cancer metabolism. 

Metabolites and their derivatives have significant potential to serve as indicative therapeutic agents due to their crucial role in the signaling, regulation, and biosynthetic roles in cells, besides having low toxicity effects compared to drugs. Dhakshinamoorthy et al. applied a GC×GC–TOFMS metabolomic-based approach to elucidate the potential mechanism of menaquinone (vitamin K2), a putative anti-leukemic metabolite [147]. This study analyzed the metabolite profiles of a leukemia model cell line (Jurkat) and non-cancerous lymphoblast cells in response to menaquinone and two common chemotherapeutics: docetaxel and doxorubicin. The findings showed that 27 out of 33 metabolites were able to discriminate between menaquinone-treated and chemotherapeutic-treated Jurkat cells, while no significant changes were observed in the non-cancerous lymphoblast cells. PCA revealed that the metabolites in menaquinone-treated samples were not correlated with those in chemotherapeutic-treated Jurkat cells, implying the unique anti-proliferative activities of menaquinone. Notably, the levels of phosphoethanolamine were found to be significantly increased in menaquinone-treated Jurkat cells, suggesting a connection between these two metabolites in displaying antileukemic activity. The application of GC×GC expands the future prospects of metabolite-based therapeutics by identifying and characterizing metabolites with potential anticancer activities, which could eventually pave the way for the discovery of novel chemotherapeutic drugs.

**Figure 3:**
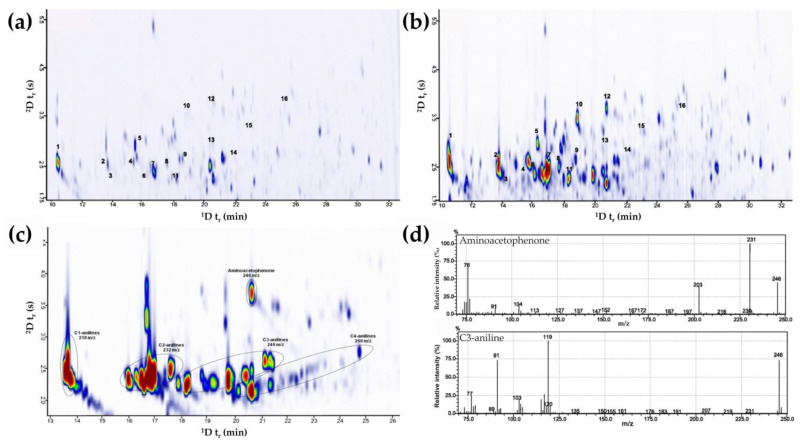
Representative GC×GC chromatograms displaying the presence of aromatic amine derivatives in: (**a**) non-smoker’s urine; (**b**) smoker’s urine; (**c**) smoker’s urine with isomeric homologs of aromatic amines labeled as C1- to C4-anilines and aminoacetophenone (**d**) mass spectra of iodinated derivatives of aminoacetophenone and C3-aniline. Adapted with permission from Ref. [148]. Copyright © 2023 by the authors; Springer Nature Group, Berlin, Germany.

Urological cancer, or urinary bladder cancer, is reported as the ninth most prevalent malignant disease and the thirteenth leading cause of cancer-related death globally [163]. An elevated risk of bladder cancer among smokers has been reported due to the presence of a potent class of carcinogen known as aromatic amines in tobacco smoke [164]. Aromatic amines are well-absorbed across and into the biological membranes through the skin, gastrointestinal and respiratory tracts, which could lead to transitional-cell carcinoma, which is known as one of the most common types of bladder cancer [165]. In-situ derivatization solid-phase microextraction (SPME) coupled with GC×GC–TOFMS was applied to determine the presence of aromatic amines in the urine of smokers and non-smokers [148]. Derivatization of aromatic amines to their corresponding aromatic iodine derivatives with enrichment on a polydimethylsiloxane/divinylbenzene fiber gave an extraction efficiency of 65–85%. Separation of 16 aromatic amines was optimized with DB-5 as the ^1^D column while the ^2^D column was selected among BPX50 and five ionic liquid columns with different polarity: SLB IL-59 (polar), SLB IL-61 (polar), SLB IL-76 (highly polar), SLB IL-82 (highly polar), and SLB IL-100 (extremely polar). The selectivity of ionic liquid columns for the iodinated analytes was comparable with the mid-polar BPX50 column; hence the BPX50 column was selected for subsequent studies as it also provides the best separation of the analytes. A lower intensity of aromatic amines was observed in the urine of non-smokers compared to that of smokers, indicating higher exposure to the carcinogenic substance among the latter group with more than 150 aromatic amine derivatives (dominated by alkylated anilines) identified (Figure 3a–c). This study also highlighted the advantages of GC×GC–MS for the separation of compounds with the same nominal mass in complex matrixes: aminoacetophenone (*m*/*z* 246) and C3-anilines (*m*/*z* 246) (Figure 3d). However, due to the high number of compounds and larger data files in GC×GC compared to the conventional GC, data handling and analysis still pose a great challenge, particularly for quantitative analysis.

### 4.3. Coronavirus Disease (COVID-19)

At the end of 2019, the outbreak of coronavirus disease (COVID-19) caused massive disruption, and a large proportion of the population worldwide was impacted. Known as severe acute respiratory syndrome coronavirus-2 (SARS-CoV-2), the coronavirus mainly affects the respiratory system with extremely heterogeneous symptoms, ranging from those with minimal impact to significant hypoxia with acute respiratory distress syndrome [166]. COVID-19 was declared a global pandemic by WHO in 2020 following a high transmission rate of SARS-CoV-2 and a lack of pre-existing immunity that resulted in the rapid spread of the life-threatening disease from its origin to other countries around the world [167,168]. Few studies have reported the use of GC×GC in the search for disease biomarkers and understanding its pathophysiological process. 

**Figure 4:**
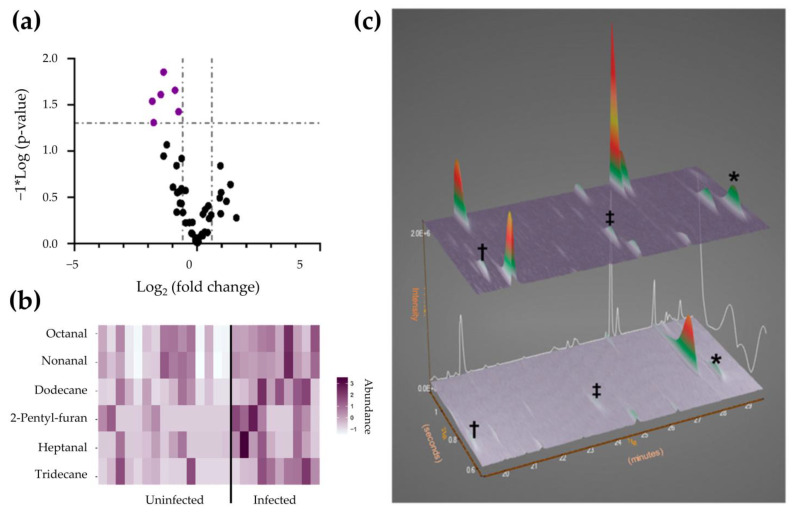
Biomarker compounds of pediatric SARS-CoV-2 infection. (**a**) Volcano plot of breath metabolites. (**b**) Heatmap visualizing the abundance of biomarkers. (**c**) GC×GC–TOFMS surface plots of representative pediatric breath samples (top, SARS-CoV-2 infected; bottom, SARS-CoV-2 uninfected; †, heptanal; ‡, octanal; *, nonanal). Adapted with permission from Ref. [150]. Copyright © 2023 by the authors; American Chemical Society, Washington, DC, United States.

Barberis et al. performed untargeted metabolomic and lipidomic profiling of 161 plasma samples from patients with pneumonia and/or respiratory failure [149]. Based on the metabolomic approach using GC×GC–TOFMS, several identified biomarkers were able to distinguish between COVID-19 and non-COVID-19 patients, critical and non-COVID-19 patients, as well as critical and non-critical COVID-19 patients. Additionally, the biomarkers were further evaluated for their diagnostic performance by examining the non-infected patients with symptoms mimicking the viral infection. The authors consequently observed a relationship between the host response to the virus and several metabolisms, inflammation, and the immune system. In another study, Berna et al. evaluated the volatile breath metabolites of 26 children (11 tested positive; 15 were negative) using thermal desorption (TD) coupled with GC×GC–TOFMS [150]. In order to ensure the quality of the breath volatiles, the level of isoprene in collected samples was evaluated as the compound is expected to present in human breath at a high concentration in comparison to that in ambient air. The authors initially selected 84 metabolites to be investigated based on previous literature. Upon analysis through volcano plot, heatmap, and contour plot, six biomarkers were found to be elevated in COVID-19-infected breath groups, discriminating them from the uninfected ones (Figure 4). It is worth mentioning that three aldehydes (octanal, nonanal, and heptanal) drew special attention as the class of compound was previously reported to be upregulated in adult breath [44]. However, the biomarkers of children’s breath are still markedly different from the adult’s breath [44] due to the stronger immune system of the younger group. From these studies [149,150], slight differences observed in the host response to infection may be attributable to factors such as age group, geographic origin of patient, and level of infection. Nevertheless, GC×GC techniques have shown to be ideally suited to improving the current understanding of important metabolic pathways and metabolisms to unearth novel therapies and upgraded control measures.

A recent study by Barberis et al. analyzed the exhaled breath condensate of COVID-19 patients and healthy controls using GC×GC–TOFMS [151]. The use of PLS-DA revealed significant differences between both groups, supported by VIP scores that presented the most prominent molecules influencing the phenotypic variances in COVID-19 exhaled breath condensate. Upon subjecting the data to further statistical analyses, 26 molecules were found to be significantly different between the infected and healthy patients; eight of them were monoglycerides of fatty acids. In the same year, Barberis et al. investigated the exposure of 51 healthcare workers to an environment with a similar probability of contracting COVID-19 via untargeted GC×GC–TOFMS metabolomic profiling of serum [152]. Initially, the workers tested negative during the initial blood collection, and 24 of them developed the disease within the next 21 days. The study revealed significant modulation of monolaurin (monoglyceride of lauric acid), oleic acid, and cholesterol between protected and predisposed subjects, suggesting their potential as COVID-19 prediction biomarkers. Notably, a two-fold increase in the level of monolaurin (monoglyceride of lauric acid) was found in subjects protected from SARS-CoV-2; meanwhile, a higher level of cholesterol was observed in subjects infected by the disease. Both studies [151,152] underscore the inherent capability of GC×GC in discovering prospective molecules for the prediction of protection against SARS-CoV-2, as well as diagnosis and monitoring of COVID-19. While fatty acids have been associated with protective roles against SARS-CoV-2 infection, cholesterol potentially carries the risk of developing the disease by promoting viral replication and entry into host cells. Future studies involving the randomized controlled trial of fatty acids and a larger cohort of subjects to confirm these findings would contribute to the development of personalized medicine and the implementation of public healthcare strategies.

### 4.4. Psychiatric Disorder

Schizophrenia, bipolar disorder, and major depressive disorder are highly complex mental conditions characterized by a diverse array of symptoms, including dysfunctions in thoughts, perceptions, emotions, and behavior [169]. Consistent with behavioral and genetic studies, the noticeable phenotypic overlap suggests the presence of potential genetic factors involved in these mental disorders. Despite ongoing research, the underlying mechanisms behind these diseases are still poorly understood. Ramaker et al. conducted a comprehensive study of the post-mortem transcriptome and metabolite profile of these psychiatric disorders to uncover the underlying disease mechanism [153]. Brain tissues across three regions (anterior cingulate cortex, dorsolateral prefrontal cortex, and nucleus accumbens) were sampled from 24 healthy controls and subjects diagnosed with schizophrenia (24 subjects), bipolar disorder (24 subjects), and major depressive disorder (24 subjects). The untargeted metabolite profiling of the anterior cingulate cortex tissue performed using GC×GC–TOFMS, followed by multi-statistical analysis, identified 141 unique metabolites that best discriminate between the schizophrenia and bipolar disorder patients from the control group. Significant downregulation in γ-aminobutyric acid (GABA) levels were observed in individuals with schizophrenia and bipolar disorder, suggesting that altered gene expression in both diseases was responsible for such biochemical changes. Nonetheless, the metabolite profile of the major depressive disorder patients displayed no significant differences from the control group. The combination of transcriptomic and metabolomic analysis demonstrated in this study provides a clearer understanding of the metabolite–gene relationship within these three multigenic psychiatric disorders. 

Olanzapine has been administered as an antipsychotic drug, specifically to manage symptoms of schizophrenia and bipolar disorder, and was lauded as the most effective drug option due to its less severe side effects [170]. However, the administration of olanzapine has been reported to cause weight gain and metabolic disturbance. The systemic effects of olanzapine have been associated with hepatic metabolism; thus, metabolomic analysis using GC×GC–TOFMS was applied to simultaneously characterize several effects of olanzapine on liver tissues and plasma in the mouse model [154]. A significant increase in the peripheral concentrations of glutamate and its metabolites was observed due to the administration of olanzapine. Based on the hepatic metabolites profile, Schmidt et al. concluded that olanzapine altered the hepatic metabolism via the simultaneous activation of both catabolic AMP-activated protein kinase (AMPK) and anabolic (mammalian target of rapamycin) pathways, inducing disturbance in the glucose and lipid metabolism. The utilization of the GC×GC–TOFMS approach in this study has shown great potential in enhancing the understanding of olanzapine dysmetabolism as well as mechanisms mediating desirable therapeutic effects of the drug, which have been incomprehensible previously. These findings will definitely be useful for the future development of new approaches to improve the long-term safety and utility of the drug. 

Recently, Eshima et al. applied headspace solid-phase microextraction (HS-SPME) coupled with the GC×GC–TOFMS method to uncover the underlying mechanism of metabolic alterations due to stress in human urinary volatilome through the diurnal cortisol cycle [155]. Cortisol is a glucocorticoid released by the adrenal glands’ cortex in response to stress. Despite numerous studies reporting the association of cortisol dysregulation with mental health and mood disorders such as depression, bipolar disorder, and schizophrenia, the underlying mechanism remains unclear. The urinary volatile metabolomic profiles of 60 healthy individuals were extracted with divinylbenzene/carboxen/polydimethylsiloxane SPME fiber, revealing 14 key metabolites associated with the measured total urinary cortisol. The predictive model developed in this study has the potential to estimate the total free urinary cortisol excretion in humans by using 14 volatilomes and seven interaction terms responsible for the metabolite variability according to the criteria identified by the two-way ANOVA (i.e., gender and sample collection time). This study highlighted the future application of the GC×GC metabolomic method for psychiatric diagnostics and long-term mental health monitoring. However, targeted studies using a larger sample cohort must be considered to further validate the identity of the compounds and to assess the prediction capability and accuracy of the developed model.

## 5. Challenges of GC×GC in Moving toward Personalized Medicine

The development of high-throughput GC×GC–MS, as one of the major platforms in the emerging field of pharmacometabolomics, demonstrates great potential in providing a more comprehensive metabolic profile, replacing the current medical practices that suffer limited chemical differentiation or metabolite coverages. Pharmacometabolomics can be termed as the prediction of the outcome of a drug or xenobiotic intervention in an individual based on a mathematical model of preintervention of metabolite signatures [171]. Since the human metabolome varies between individuals (depending on the genetics, environment, and gut microbiota), personalized medicine is tailored toward particular metabolic characteristics of an individual for effective treatments. Nevertheless, there is an obstacle in translating discovered pharmacometabolomic biomarkers into clinical practice primarily due to the scarcity of large-scale validation and characterization strategies for the technique. These steps are indeed substantial in ensuring the correct characterization and selection of high-quality biomarkers. It is worth noting that these resources can be well generated by collaborative efforts between researchers, clinicians, and bioinformaticians. Recently, detailed guidelines on best reporting practices and analytical quality management for MS-based analyses have been published [110,172,173], supporting the former reporting guidelines outlined by Metabolomics Standards Initiative [174]. The standard is designed to provide confidence to the scientific community in reproducing or incorporating meaningful data into other analyses, either directly or indirectly, for the discovery of new relationships between metabolomes and biological states [67,173,175]. Additionally, the identification of unknown chemical entities (or dark matter) presents a current bottleneck in the untargeted analysis workplan [176,177,178]. Several open-access and commercial databases that comprise numerous spectral information of compounds (based on both real metabolite entries and in silico predicted spectra) have been developed [179]. In reality, however, a human metabolomic profile is much more complex where at least 500,000 compounds are (or may be) present, and each class of compound contains immense combinatorial capacity (e.g., over 40,000 different lipids can be generated from 20 types of fatty acids that are formed from six common triacylglycerides) [180]. Samples containing polar functional groups require derivatization steps to increase their volatility which further complicates the process of elucidating compound structure without any database match. These issues lead to potential information in typical metabolomics studies being lost and limit its interpretation and understanding of biological mechanisms. A commentary reported by Silva et al. in 2015 stated that a single untargeted metabolomics study could only annotate 1.8% of spectra, which signified that the majority of metabolites present in a sample are yet to be identified [176]. Therefore, there is a critical need for an improved data analysis strategy or workflow that ideally provides optimal coverage for compound identification. For the reader’s interest, a few excellent reviews have been published recently on the potential of computational approaches to facilitate metabolite identification [181,182,183]. Even though the quest for ideal biomarkers is still underway, the widespread implementation of best practice and quality management recommendations, as well as the development of cheminformatic tools for compound characterization, is envisaged to be a key component in pushing GC×GC–MS toward clinical translation and personalized medicine.

## 6. Concluding Remarks

Recent years have seen remarkable advances in GC×GC technology as a powerful tool to unravel compositions of various intricate biological and pharmaceutical samples, underpinned by substantial improvements in modulator technologies and detection systems. The most noteworthy has been the technological advances in GC×GC–HRMS where elemental formula assignment of resolved molecules (that may be comprised of ‘known unknowns’ or ‘unknown unknowns’) made possible with accurate mass measurement displaying mass uncertainties ≤5 ppm. Despite the great potential of GC×GC–HRMS in bioanalytical studies, it has yet to be fully exploited. Apart from low-resolution TOFMS, the use of the full complement of HRMS tools (e.g., accTOFMS, FTICRMS, Orbitrap, and accQTOFMS) for GC×GC studies on disease-related biomarkers and host response to drug treatments have not been explored. Perhaps this reflects the preference for GC–HRMS due to the perceived complexity and difficulties in implementing a successful GC×GC–HRMS method and the need for specialized instrumentations, software, and technical expertise. In regards to the last 10 years of progress, the application of GC×GC in drug discovery and analysis has resulted in very interesting findings that focused on the understanding of the perturbation of metabolites in response to disease progression and drug treatment. Despite the many advantages of GC×GC, critical aspects still require additional improvement and attention, as indicated by the slow uptake of this technique by bioanalysts. Many of the reviewed studies still use relative concentrations to report/compare drug concentrations and/or clinical biomarkers. Greater efforts are needed to demonstrate the reliability and reproducibility of GC×GC for absolute quantitative measurement of metabolites in complex biological samples. In particular, the entire workflow covering sample collection, derivatization, preparation, and GC×GC–MS should be validated according to the criteria and guidelines defined for bioanalytical methods. Additionally, the assessment of its robustness for analysis of large biological sample sets over a long period of time has yet to be determined. To further drive the utility of GC×GC–HRMS for pharmacometabolomics studies, complete automation of chromatographic pre-processing and statistical analysis tools are suggested, which can benefit inexperienced users in extracting useful biological information from the high-dimensional raw data. The integration of fully automated artificial intelligence-driven GC×GC–HRMS approaches for untargeted metabolomics are still in its infancy, but notable efforts are being made, and if this is successful, GC×GC–HRMS might become the next workhorse in bioanalytical-related studies offering unprecedented resolving power, translated to enhanced identification. Nevertheless, it is anticipated that the implementation of GC×GC in drug discovery and analysis will continue to grow and become a realistic option for the future development of personalized disease diagnosis, patient monitoring, and treatment response evaluation.

## Figures and Tables

**Table 1:** Summary of GC×GC strategies in the field of drug discovery and analysis.

Disease	Target/Study	Matrix	Sample Preparation and Derivatization	Stationary-Phase Combination	Modulator	Detector	Ref.
Tuberculosis	Isoniazid resistance in *Mycobacterium tuberculosis*	*M. tuberculosis* strain	Liquid extraction with CHCl_3_/MeOH/water (1:3:1) followed by derivatization with MXHCl in pyridine and BSTFA with 1% TMCS.	^1^D: Rxi-5Sil MS (30 m × 0.25 mm ID × 0.25 μm *d*_f_) ^2^D: Rxi-17 MS (1.0 m × 0.10 mm ID × 0.10 μm *d*_f_)	Dual-stage cryogenic modulator ^a^	TOFMS	[138]
	Metabolomic profiling of Tb and non-Tb patients	Urine	Liquid extraction with ethyl acetate, followed by diethyl ether. Sample was derivatized with BSTFA, TMCS, and pyridine.	^1^D: Rxi-5Sil MS (30 m × 0.25 mm ID × 0.25 μm *d*_f_) ^2^D: Rxi-17Sil MS (0.9 m × 0.10 mm ID × 0.10 μm *d*_f_)	Cryogenic modulator ^a^	TOFMS	[139]
	Characterization of successful and failed Tb treatment outcomes	Urine	Liquid extraction with ethyl acetate, followed by diethyl ether. Sample was derivatized with BSTFA, TMCS, and pyridine.	^1^D: Rxi-5Sil MS (30 m × 0.25 mm ID × 0.25 μm *d*_f_) ^2^D: Rxi-17 MS (0.9 m × 0.10 mm ID × 0.10 μm *d*_f_)	Cryogenic modulator ^a^	TOFMS	[140]
	Prediction of Tb treatment failure at the time of diagnosis	Urine	Liquid extraction with ethyl acetate, followed by diethyl ether. Sample was derivatized with BSTFA, TMCS, and pyridine.	^1^D: Rxi-5Sil MS (30 m × 0.25 mm ID × 0.25 μm *d*_f_) ^2^D: Rxi-17 MS (0.9 m × 0.10 mm ID × 0.10 μm *d*_f_)	Cryogenic modulator ^a^	TOFMS	[141]
	Antimicrobial mechanisms of colistin sulfate on *M. tuberculosis*	*M. tuberculosis* strain	Liquid extraction with CHCl_3_/MeOH/water (1:3:1) followed by sample derivatization with MSTFA and 1% TMCS.	^1^D: Rxi-5Sil MS (30 m × 0.25 mm ID × 0.25 μm *d*_f_) ^2^D: Rxi-17 MS (1.2 m × 0.25 mm ID × 0.25 μm *d*_f_)	NA ^b^	TOFMS	[142]
	Metabolic profiling of *M. tuberculosis* in the presence and absence of ciprofloxacin	*M. tuberculosis* strain	Liquid extraction with CHCl_3_/MeOH/water (1:3:1) followed by derivatization with MXHCl in pyridine and BSTFA with 1% TMCS.	^1^D: Rxi-5Sil MS (28.8 m × 0.25 mm ID × 0.25 μm *d*_f_) ^2^D: Rxi-17 MS (1.2 m × 0.25 mm ID × 0.25 μm *d*_f_)	NA ^b^	TOFMS	[143]
	Antimycobacterial mechanism of decoquinate derivative RMB041	*M. tuberculosis* strain	Liquid extraction with CHCl_3_/MeOH/water (1:3:1) followed by derivatization with MXHCl in pyridine and BSTFA with 1% TMCS.	^1^D: Rxi-5Sil MS (28.8 m × 0.25 mm ID × 0.25 μm *d*_f_) ^2^D: Rxi-17 MS (1.2 m × 0.25 mm ID × 0.25 μm *d*_f_)	NA ^b^	TOFMS	[144]
Cancer	Effect of docetaxel treatment on ovarian cancer cells and stem cells	OVCAR-3 ovarian cancer cell and isogenic ovarian cancer stem cell	Extracts were dried using vacuum concentrator and derivatization with OMXHCl in pyridine, MSTFA, and 1% TMCS.	^1^D: HP-5 (30 m × 0.32 mm ID × 0.25 μm *d*_f_) ^2^D: Rtx-200 (2 m × 0.25 mm ID × 0.25 μm *d*_f_)	NA ^b^	TOFMS	[145]
	Metabolic differences between ovarian cancer cells and their stem cells	OVCAR-3 ovarian cancer cell and isogenic ovarian cancer stem cell	Extracts were dried using vacuum concentrator and derivatized with OMXHCl in pyridine, MSTFA, and 1% TMCS.	^1^D: HP-5 (30 m × 0.32 mm ID × 0.25 μm *d*_f_) ^2^D: Rtx-200 (2 m × 0.25 mm ID × 0.25 μm *d*_f_)	NA ^b^	TOFMS	[146]
	Antiproliferative role of menaquinone (vitamin K2) on the leukemic Jurkat cell line	Jurkat and lymphoblast cells	Extracts were dried using vacuum concentrator and derivatized with OMXHCl in pyridine, MSTFA, and 1% TMCS.	^1^D: HP-5 (30 m × 0.320 mm ID × 0.25 μm *d*_f_) ^2^D: Rtx-200 (2 m × 0.25 mm ID × 0.25 μm *d*_f_)	NA ^b^	TOFMS	[147]
	Aromatic amines in smokers’ urine	Urine volatiles	Liquid extraction with diethyl ether followed by back extraction with concentrated hydrochloric acid (37 %). Samples were derivatized through diazotization and iodination. Derivatized analytes were extracted by HS-SPME method with PDMS/DVB fiber.	^1^D: DB-5 (30 m × 0.25 mm ID × 0.25 μm *d*_f_) ^2^D: BPX50 (2.7 m × 0.15 mm ID × 0.15 μm *d*_f_)	Cryogenic modulator ^a^	TOFMS	[148]
Coronavirus disease	Large-scale metabolomic profiling	Plasma	Liquid extraction with ACN/IPA/water (3:3:2) solution. Sample was derivatized with MOX and BSTFA.	^1^D: Rxi-5Sil MS (30 m × 0.25 mm ID × 0.25 μm *d*_f_) ^2^D: Rxi-17Sil MS (2 m× 0.25 mm ID × 0.25 μm *d*_f_)	LECO QuadJet thermal modulator	TOFMS	[149]
	Targeted metabolite changes in children with SARS-CoV-2 infection	Breath samples	Collection of breath samples into SamplePro FlexFilm sample bag, then transferred into thermal desorption sorbent tubes.	^1^D: Stabilwax (30 m × 0.25 mm ID × 0.25 μm *d*_f_) ^2^D: Rtx-200 MS (5 m × 0.25 mm ID × 0.10 μm *d*_f_)	Flow modulator^a^	TOFMS	[150]
	Untargeted metabolomic profiling of COVID-19 and non-COVID-19 patients	Exhaled breath condensate	Liquid extraction with ACN/IPA/water (3:3:2) solution. Sample was derivatized with MOX and BSTFA.	^1^D: Rxi-5Sil MS (30 m × 0.25 mm ID × 0.25 μm *d*_f_) ^2^D: Rxi-17Sil MS (2 m× 0.25 mm ID × 0.25 μm *d*_f_)	LECO QuadJet thermal modulator	TOFMS	[151]
	Untargeted metabolomics profiling to predict protection against infection	Serum	Liquid extraction with ACN/IPA/water (3:3:2) solution. Sample was derivatized with MOX and BSTFA.	^1^D: Rxi-5Sil MS (30 m × 0.25 mm ID × 0.25 μm *d*_f_) ^2^D: Rxi-17Sil MS (2 m × 0.25 mm ID × 0.25 μm *d*_f_)	LECO QuadJet thermal modulator	TOFMS	[152]
Psychiatric disorder	Post-mortem molecular profiling of three psychiatric disorders	Brain tissue	Frozen brain tissue was homogenized in 50% MeOH and dried using vacuum concentrator. Derivatization was carried out with OMX in pyridine and MSTFA.	^1^D: Rtx-5MS (20 m × 0.25 mm × 0.5 μm *d*_f_) ^2^D: Rtx-200MS (2.5 m × 0.18 mm × 0.2 μm *d*_f_)	Cryogenic modulator ^a^	TOFMS	[153]
	Olanzapine side effects on hepatic metabolism	Mice liver and plasma	Liquid extraction with ice-cold MeOH followed by drying with centrifugal evaporator and dissolved in ACN. Sample derivatization was carried out with MTBSTFA and 1% TBDMSCI.	^1^D: DB-5MS (60 m× 0.25 mm ID × 0.25 μm *d*_f_) ^2^D: DB-17MS (1 m × 0.25 mm ID × 0.25 μm *d*_f_)	LECO QuadJet thermal modulator	TOFMS	[154]
	Dysregulation of cortisol secretion	Urine volatiles	Urine volatiles were extracted by HS-SPME method with DVB/CAR/PDMS-coated fiber.	^1^D: Rxi-624 Sil MS (60 m × 0.25 mm × 1.4 µm *d*_f_) ^2^D: Stabilwax (1 m × 0.25 mm × 0.5 µm *d*_f_)	QuadJet cryogenic modulator ^a^	TOFMS	[155]

^a^ Detailed information not available. ^b^ No information available. Abbreviations: ACN: acetonitrile; BSTFA: N,O-bis(trimethylsilyl) trifluoroacetamide; CAR: Carboxen; CHCl_3_: chloroform; COVID-19: Coronavirus disease; DVB: divinylbenzene; HS–SPME: head-space solid-phase microextraction; IPA: isopropyl alcohol; MeOH: methanol; MOX: methoxyamine; MSTFA: N-methyl-N-trimethylsilyl) trifluoroacetamide; MTBSTFA: N-(tertbutyldimethylsilyl)-N-methyltrifluoroacetamide; MXHCl: methoxamine hydrochloride; OMX: O-methoxylamine; OMXHCl: O-methylhydroxylamine hydrochloride; PDMS: polydimethylsiloxane; Tb: Tuberculosis; TBDMSCI: tert-butyldimethylchlorosilane; TMCS: trimethylsilyl chloride; TOFMS: time-of-flight mass spectrometry.

## Data Availability

Not applicable.
